# The two therapeutic strategies of surgical intervention and medical management in a patient with enhanced-fibrinolytic type of disseminated intravascular coagulation after aortic replacement for Stanford type A aortic dissection with chronic heart and renal failure

**DOI:** 10.1186/s12872-024-03750-0

**Published:** 2024-01-29

**Authors:** Hiroki Okamoto, Tomoya Ozawa, Tomoaki Suzuki, Yoshihisa Nakagawa

**Affiliations:** 1 Department of Internal Medicine, Division of Cardiovascular Medicine, Shiga University of Medical Science, Otsu, 520-2192 Japan; 2 Department of Surgery, Division of Cardiovascular Surgery, Shiga University of Medical Science, Otsu, Japan

**Keywords:** Disseminated intravascular coagulation, Aortic disease, Hematoma, Anticoagulant therapy, Antifibrinolytic therapy

## Abstract

**Background:**

Management of the enhanced-fibrinolytic type of disseminated intravascular coagulation (DIC) caused by aortic disorders is the two strategies of surgical intervention and medical treatment based on the patient’s age and comorbidities.

**Case presentation:**

An 81-year-old woman with a history of two previous aortic surgeries and chronic heart and renal failure was admitted for uncontrollable subcutaneous hemorrhage. The hemorrhage was caused by the enhanced-fibrinolytic type of disseminated intravascular coagulation (DIC) caused by periprosthetic graft hematoma after aortic replacement for Stanford type A aortic dissection. Open thoracic hemostasis temporarily controlled the subcutaneous hemorrhage, but she was readmitted for the recurrence seven months after discharge. On the second admission, the combination of anticoagulant and antifibrinolytic agents was successful.

**Conclusion:**

Management of the enhanced-fibrinolytic type of DIC caused by aortic disorders is important of a successful combination of surgical and medical therapy tailored the patient’s condition.

## Background

Disseminated intravascular coagulation (DIC) is systemic coagulant disorder caused by underlying diseases, such as severe infection, malignant tumor, liver failure, and vascular disorders. This syndrome is characterized by bleeding tendency with consumption of platelets and coagulation factors, and by multiple organ failure with microvascular thrombosis [1, 2]. DIC is classified into three types by the degree of fibrinolytic activation: suppressed-fibrinolytic type, balanced-fibrinolytic type, and enhanced-fibrinolytic type. Enhanced-fibrinolytic type DIC, which is associated with marked fibrinolytic activation, have been reported in acute promyelocytic leukemia (APL) and aortic disorders such as aortic aneurysm and dissection [3]. Especially, DIC associated with aortic disorders progress chronically in no symptom, but some invasions can induce a decompensated state with hemorrhagic complication [4]. The optimal treatment for DIC associated with aortic disorders is surgical intervention, however it is not often suitable due to the patient’s medical condition such as advanced age or severe comorbidities [2, 5]. Several case reports showed that medical treatments, such as anticoagulant agents, antifibrinolytic agents, and a combination of both, were effective for inoperable patients with DIC associated with aortic disorders [4, 6–13]. We describe a case in which two strategies of surgical intervention and medical treatment were effective for the enhanced-fibrinolytic type DIC after aortic replacement for Stanford type A aortic dissection with chronic heart and renal failure.

### Case presentation

**Fig. 1:**
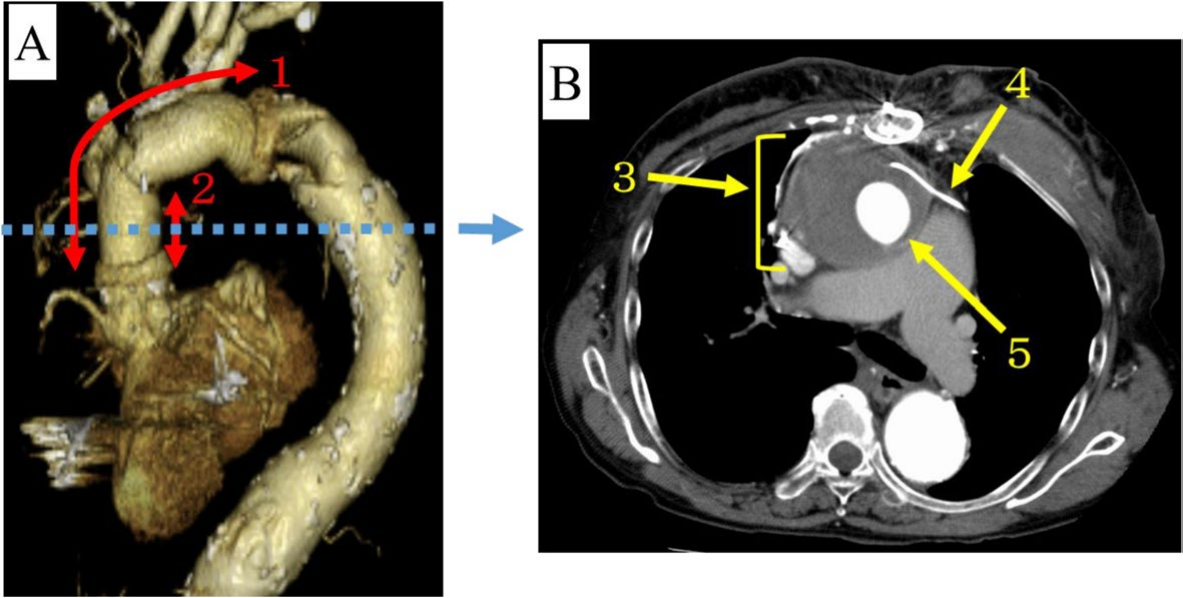
Enhanced computed tomography. **A**: 3D imaging from left anterior oblique; ①Aortic arch replacement on the first sternotomy. ②Ascending aorta replacement on the second sternotomy. **B**: Axial view; ③Periprosthetic vascular graft hematoma ④Lead on epicardium. ⑤Prosthetic vascular graft on ascending aorta

The patient was an 81-year-old woman with left ventricular dysfunction (left ventricular ejection fraction, 49%) and severe chronic kidney disease (Estimated glomerular filtration rate, 10.6 mL/min/1.73m^2^). She was on warfarin due to long-standing atrial fibrillation (AF). She underwent total arch replacement for Stanford type A acute aortic dissection at age of 70 (Fig. 1A). During the sternotomy, an epicardial left ventricular lead was placed because she was previously diagnosed as dilated cardiomyopathy with left ventricular ejection fraction of 33% and left bundle branch block. She subsequently underwent cardiac resynchronization therapy-defibrillator (CRT-D) implantation using this lead. Thereafter, a pseudoaneurysm developed around the anastomosis of the prosthetic vascular graft on the proximal side and continued to growth over time. Therefore, she underwent ascending aortic replacement at age of 76 (Fig. 1A). Although hematoma developed around the prosthetic vascular graft on the ascending aorta one year after the second sternotomy, she followed an uneventful course without symptoms for several years (Fig. 1B).

### The first admission for hematoma around the CRT-D

**Fig. 2:**
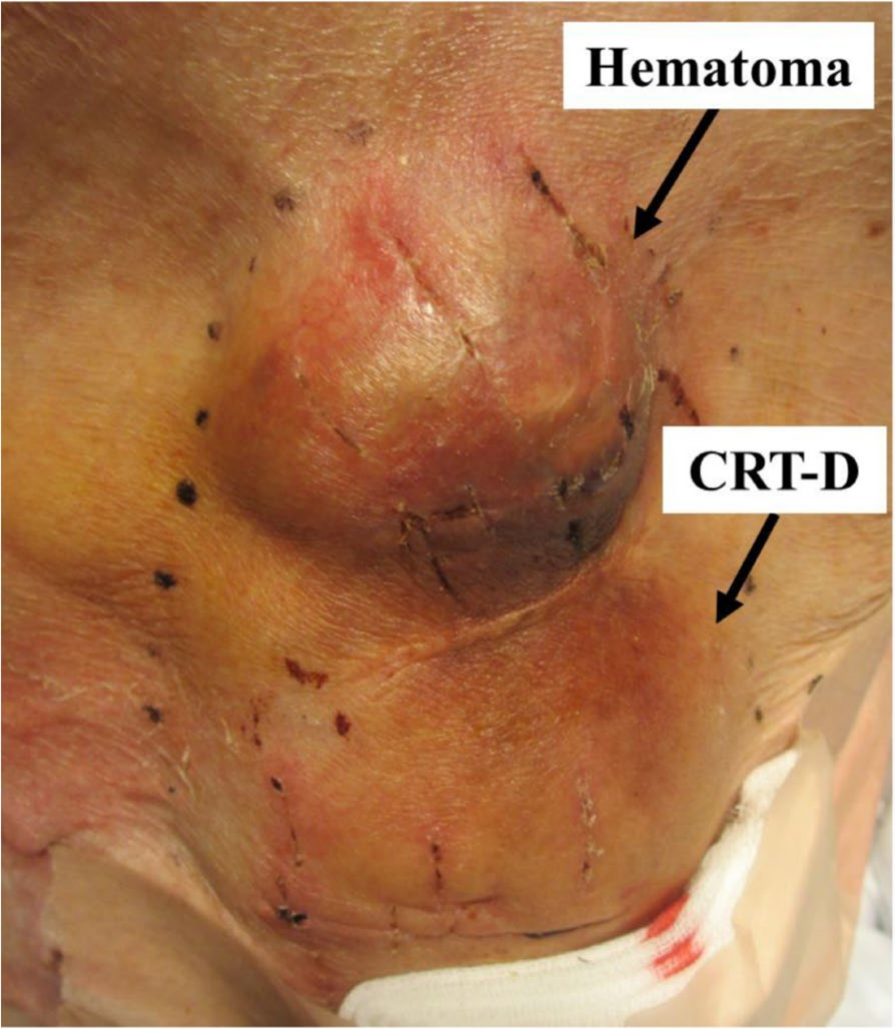
The picture of hematoma around cardiac resynchronization therapy-defibrillator

**Fig. 3:**
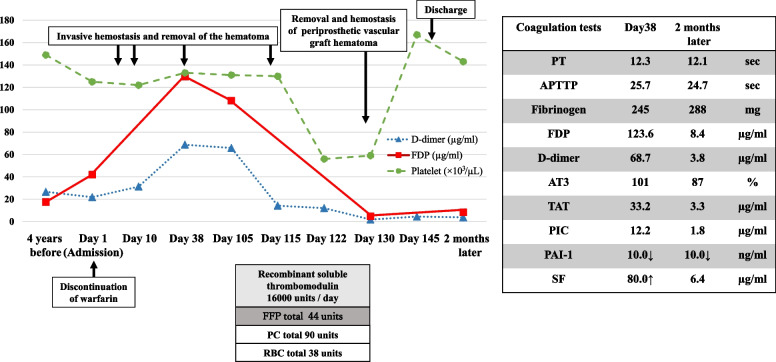
Clinical course of the first admission. The hematoma around the CRT-D was not controlled with invasive procedures and recombinant soluble thrombomodulin. After removal and hemostasis of the periprosthetic vascular graft hematoma, the hematoma around the CRT-D did not recur and the DIC improved. APTTP, activated partial thromboplastin time; AT3, antithrombin III; CRT-D, cardiac resynchronization therapy-defibrillator; DIC, disseminated intravascular coagulation; FDP, fibrinogen degradation products; FFP, fresh frozen plasma; PAI-1, plasminogen activator inhibitor-I; PC, platelet concentrate; PIC, plasmin-α2-plasimin inhibitor complex; PT, prothrombin time; RBC, red blood cell; SF, soluble fibrin; TAT, thrombin-antithrombin complex

Four years after the second sternotomy, she was admitted because hematoma developed around CRT-D on left greater pectoral muscle (Fig. 2). Warfarin was discontinued, but this hematoma subsequently increased. Despite several invasive treatment of hemostasis and removal of the hematoma, the hematoma recurred within days on each time. Because laboratory data revealed a low platelet count and excessive levels of coagulation and fibrinolytic factors [platelet count 11.3×10^4^/µL; fibrinogen, 245mg/dL; fibrinogen degradation products (FDP), 123.6 µg/ml; D-dimer, 68.7 µg/ml; antithrombin III (ATIII), 101%; thrombin-antithrombin complex (TAT), 33.2 ng/ml; plasmin-α2-plasimin inhibitor complex (PIC), 12.2 µg/ml; plasminogen activator inhibitor-I (PAI-I), <10.0 ng/ml; soluble fibrin (SF), >80.0 µg/ml], we suspected that the hematoma may have resulted from the enhanced-fibrinolytic type of DIC induced by the periprosthetic vascular graft hematoma on the ascending aorta. Intravenous administration of recombinant soluble thrombomodulin 16000 units were initiated and fresh frozen plasma and platelet concentrate were appropriately administered. When the DIC had improved, we again performed invasive hemostasis and removal of the hematoma around the CRT-D. However, the hematoma recurred. Therefore, we determined to perform the sternotomy for the removal of periprosthetic vascular graft hematoma, even though she had very high risks for sternotomy due to a history of two previous sternotomy, chronic heart failure, and severe chronic kidney disease. At the sternotomy, we found massive organized thrombus around prosthetic vascular grafts and pulsatile bleeding from the anastomotic portion on the proximal side. We were able to suture the anastomotic portion on the proximal side by monofilament suture with a felt. After the sternotomy, the hematoma around CRT-D did not recur and the DIC improved (Fig. 3: FDP: 8.4 µg/ml, D-dimer: 3.8 µg/ml, TAT:3.3 µg/ml, PIC: 12.2 µg/ml). She had an uneventful post-operative course and was discharged 19 days later from the sternotomy.

### The second admission for hematoma around the CRT-D

**Fig. 4:**
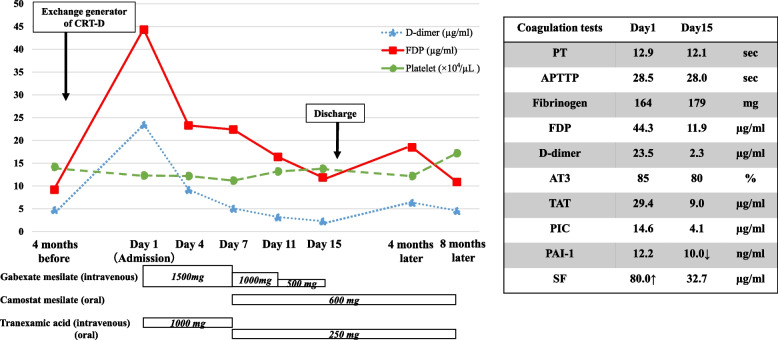
Clinical course of the second admission. After replacement of the CRT-D generator, the hematoma around the CRT-D recurred and the DIC worsened. DIC improved with the administrate of gabexate mesylate and tranexamic acid. DIC didn’t worsen after switching to oral agents. APTTP, activated partial thromboplastin time; AT3, antithrombin III; CRT-D, cardiac resynchronization therapy-defibrillator; DIC, disseminated intravascular coagulation; FDP, fibrinogen degradation products; PAI-1, plasminogen activator inhibitor-I; PIC, plasmin-α2-plasimin inhibitor complex; PT, prothrombin time; SF, soluble fibrin; TAT, thrombin-antithrombin complex

After discharge, she had no hemorrhagic complications, although a simple CT showed the new high absorptive areas of suspected hematoma around prosthetic vascular grafts one month after the third sternotomy. Three months after the third sternotomy, she needed to replace the CRT-D generator for battery depletion. We performed the generator replacement with caution for bleeding, and fortunately she could be discharged early after the surgery for no recurrence of hematoma around the CRT-D. However, four months after the generator replacement, she was admitted for recurrence of hematoma around the CRT-D. In addition, laboratory data again showed excessive levels of coagulation and fibrinolytic factors (Fig. 4: FDP, 44.3 µg/ml; D-dimer, 23.5 µg/ml; TAT, 6.9 µg/ml; PIC, 14.6 µg/ml). Because the hematoma was not controlled with recombinant soluble thrombomodulin on the first admission, we discussed the medical management strategy. We determined to administer a protease inhibitor and an antifibrinolytic agent. We initiated continuous intravenous infusion of gabexate mesilate 1500 mg and tranexamic acid 1000 mg per daily. Thereafter, the hematoma did not increase, and the coagulation and fibrinolytic factors had improved. We switched oral agents of camostat mesilate 600 mg and tranexamic acid 500 mg daily on day 7 after admission. The dose of continuous intravenous infusion of gabexate mesilate was gradually reduced from day 7. Continuous intravenous infusion of gabexate mesilate was discontinued on day 15. Coagulation and fibrinolytic factors didn’t deteriorate after switching to oral agents (Fig. 4: FDP: 11.9 µg/ml, D-dimer: 2.3 µg/ml, TAT: 9.0 µg/ml, PIC: 4.1 µg/ml). Therefore, she could be discharge on day 20. She was followed up uneventfully as an outpatient in our hospital after discharge. The DIC was controlled with oral agents, and the hematoma around the CRT-D gradually decreased and disappeared about one month after discharge. However, repeated hospitalizations led to her progressive dementia and frail. She died of renal failure about one year after discharge.

## Discussion and conclusion

Enhanced-fibrinolytic type DIC is characterized by marked fibrinolysis activation corresponding to coagulant activation. This type of DIC has been reported in APL and aortic disorders such as aneurysm and dissection. The mechanism of DIC differs depending on the underlying disease. In APL, abnormally high levels of expression of annexin II on APL cells induces marked fibrinolytic activation [14]. On the other hand, DIC associated with aortic disorders is caused by excessive consumption of coagulation factors due to endothelial disruption with atheromatous plaque or dissection of the aorta [4, 11]. DIC associated with aortic disorders decompensates when triggered by trauma or an invasive procedure such as tooth extraction triggers, resulting in hemorrhagic complications that are difficult to control. In this case, we assumed that chronic DIC was caused by excessive consumption of coagulation factors due to hematoma leakage from prosthetic vascular grafts and some incident triggered the hematoma around the CRT-D as hemorrhagic complications [4].

The optimal treatment for DIC is the treatment for the underlying disease. Hence, surgical intervention is the optimal treatment for DIC associated with aortic disorders. However, it is often difficult due to the patient’s medical condition such as advanced age or severe comorbidities [2, 5]. Several anticoagulant agents have been reported to be effective for DIC, such as heparin [6], synthetic protease inhibitors [7, 8], recombinant soluble thrombomodulin [9], and direct oral anticoagulants [4, 10]. Antifibrinolytic agents such as tranexamic acid are generally not recommended for the DIC due to the increased risk of thromboembolic complications, but it could be a reasonable option in combination with anticoagulant agents for DIC with marked activation of fibrinolysis [2, 11–13, 15]. In the first admission of this case, we performed the third sternotomy as the treatment for the underlying disease because the hematoma around the CRT-D was not controlled despite the administration of recombinant soluble thrombomodulin. However, the hematoma might improve spontaneously because D-dimer had been decreased in association with administration of the agent, if we continued the administration without invasive procedures on the hematoma. On the contrary, multiple invasive procedures on the hematoma might lead to a worsening of the DIC. The recurrence of DIC leading to the second admission may also have been triggered by the invasive procedure of the CRT-D replacement. In the second admission, we could control the hematoma by the combination of synthetic protease inhibitor and tranexamic acid. We chose gabexate mesilate of synthetic protease inhibitor concerned about hyperkalemia of the side effect of nafamostat mesilate because she had severe renal dysfunction, although there were no reports of gabexate mesilate for DIC associated with aortic disorders to the best of our knowledge. Because gabexate mesilate had less antifibrinolytic activity than nafamostat mesilate, we determined to add tranexamic acid [16]. Subsequently, in contrast to the first admission, we could observe a reduction of the hematoma without invasive procedures after decreasing the coagulation markers of D-dimer and FDP with the medical therapy. In addition, by continuing a synthetic protease inhibitor and tranexamic acid as oral agents after discharge, we were able to suppress the hemorrhagic complication in the long term. We did not switch to an AF-adapted DOAC because she had severe renal dysfunction. We believed that the long-term use of these agents was reasonable because of recurrent hemorrhagic events.

In summary, we experienced two different strategies for enhanced-fibrinolytic type DIC with the hemorrhagic complication in one case. This case showed the importance of a successful combination of surgical and medical therapy for hemorrhagic complications due to enhanced-fibrinolytic type DIC.

## Data Availability

All relevant data supporting the conclusions of this article are included within the article.

## Abbreviations

AF: Atrial fibrillation
APL: Acute promyelocytic leukemia
CRT-D: Cardiac resynchronization therapy-defibrillator
DIC: Disseminated intravascular coagulation
